# Evaluating the Turkey as a novel model for arthroscopic knee surgery research: a comparative analysis with canine and rabbit

**DOI:** 10.1186/s12891-025-09098-6

**Published:** 2025-09-02

**Authors:** Kai-Lan Hsu, Po-Yen Ko, Katy L. Lydon, Weihong Zhu, Shunen Xu, Ramona L. Reisdorf, Chunfeng Zhao

**Affiliations:** 1 Mayo Clinic, Rochester, MN USA; 2 Department of Orthopedics, National Cheng Kung University Hospital, College of Medicine, National Cheng Kung University, Tainan, Taiwan

**Keywords:** Animal model, Knee, ACL, PCL, Meniscus

## Abstract

**Background:**

Animal models are crucial for developing treatments for knee injuries. This study compared knee joint anatomy and arthroscopic surgery feasibility among turkeys, canines, and rabbits.

**Methods:**

Knee samples from cadavers of turkeys, canines, and rabbits (*n* = 18 per group) were examined to compare anatomical, histological, and biomechanical characteristics across species. Detailed measurements were taken of the bony structures, ligaments, and menisci, while histological analyses focused on the meniscus and cartilage. Biomechanical testing of the meniscus was also conducted. Additionally, the feasibility of performing diagnostic arthroscopy, cranial cruciate ligament transection, and total medial meniscectomy was assessed and compared between species and surgeons.

**Results:**

Significant differences were observed in various anatomical structures among the three species, with turkeys having a significantly wider patella and a different cruciate ligament orientation. Histological analysis showed substantial differences in meniscus structure and cartilage thickness between species. However, the biomechanical properties of the meniscus were fairly comparable across species. There was no significant difference in surgical time among surgeons performing these arthroscopic procedures. Nevertheless, surgery took significantly longer in rabbits than in turkeys or canines, while no significant difference in duration was found between turkeys and canines.

**Conclusions:**

This study demonstrates the anatomical, histological, and biomechanical properties, as well as the feasibility of arthroscopic surgery, in three potential animal models of the knee joint. Results suggest the turkey may serve as a promising bipedal animal model for investigational arthroscopic knee surgery and research on associated traumatic knee injuries. However, anatomical differences from the human knee require careful evaluation of its suitability as a simulator before research.

**Supplementary Information:**

The online version contains supplementary material available at 10.1186/s12891-025-09098-6.

## Introduction

The incidence of knee soft tissue injuries, such as cruciate ligament tears, meniscal damage, and cartilage lesions, has significantly increased over the past two decades [1–3], often leading to acute post-traumatic arthritis [4, 5]. Effective treatments are needed, but thorough preclinical evaluation in animal models is essential to ensure efficacy and safety [6]. These models provide a controlled setting for developing and assessing novel therapies [7–9]. sheep [10], porcines [11], canines [12], and rabbits [13] have been used to model human knee pathology, but anatomical and biomechanical differences pose challenges.

Furthermore, arthroscopic surgery has been increasingly utilized in large animal studies s, such as canine [14], minipig [15] and sheep [16], providing a minimally invasive approach for diagnostics and interventions while preserving joint integrity. However, the use of large animal models remains costly and time-consuming, with slow osteoarthritis (OA) progression [17]. Therefore, identifying cost-effective, short-lived models suitable for arthroscopic knee surgery is essential for advancing translational research.

Avians, a kind of bipedal species, might have some similarity with humans. Studies conducted by Guo et al. have revealed striking similarities in hip joint biomechanics between humans and emus [18]. Additionally, Kadar et al. demonstrated that turkey flexor tendons possess many comparable properties to human flexor tendons, and Sabbagh et al. reported the turkey digit anatomy similarly to human’s [19, 20]. Toyoshima et al. demonstrated that turkeys are a viable animal model for lower extremity compartment syndrome studies [21]. Given that turkeys are bipedal animals, they offer a unique opportunity to investigate periarticular knee injuries and potentially provide more directly applicable results. The larger size of turkeys, in comparison to some other avian species, and their relatively lower maintenance costs further enhance their potential as an animal model for arthroscopic knee studies.

Currently, there is a lack of research on the specific structure, biomechanics, and application of arthroscopy in turkey knees. Therefore, this study aims to (1) investigate the anatomical, mechanical, and histological properties of turkey knees, comparing them with established animal models such as canines and rabbits, and (2) compare the feasibility of conducting arthroscopic examinations, cranial cruciate ligament transection, and total medial meniscectomy in turkey knees with canine and rabbit knees. We hypothesize that the turkey knee is a viable alternative model for arthroscopic knee surgery research.

## Materials and methods

### Research model

Cadaveric knee samples were harvested from fresh turkeys (*n* = 18, 18 pairs), canines (*n* = 18, 18 pairs) and rabbits (*n* = 18, 18 pairs). These two species were selected for comparison because they are commonly used as models for knee-related surgeries and are similar in size to turkeys. All cadaveric knees were obtained following euthanasia from other Institutional Animal Care and Use Committee (IACUC) approved protocols. Six pairs of legs in each species were used for anatomical measurements, histological, and biomechanical studies. Another twelve pairs of legs in each species were used for arthroscopic surgery. In turkeys, there were nine males weighing 8–10 kg and nine females weighing 6–8 kg aged 10–12 months. In canines, there were nine male and nine females all weighting 30–35 kg aged 1 year. In rabbit, there were nine males and nine females all weighing 6–8 kg with aged 4–5 months.

**Fig. 1:**
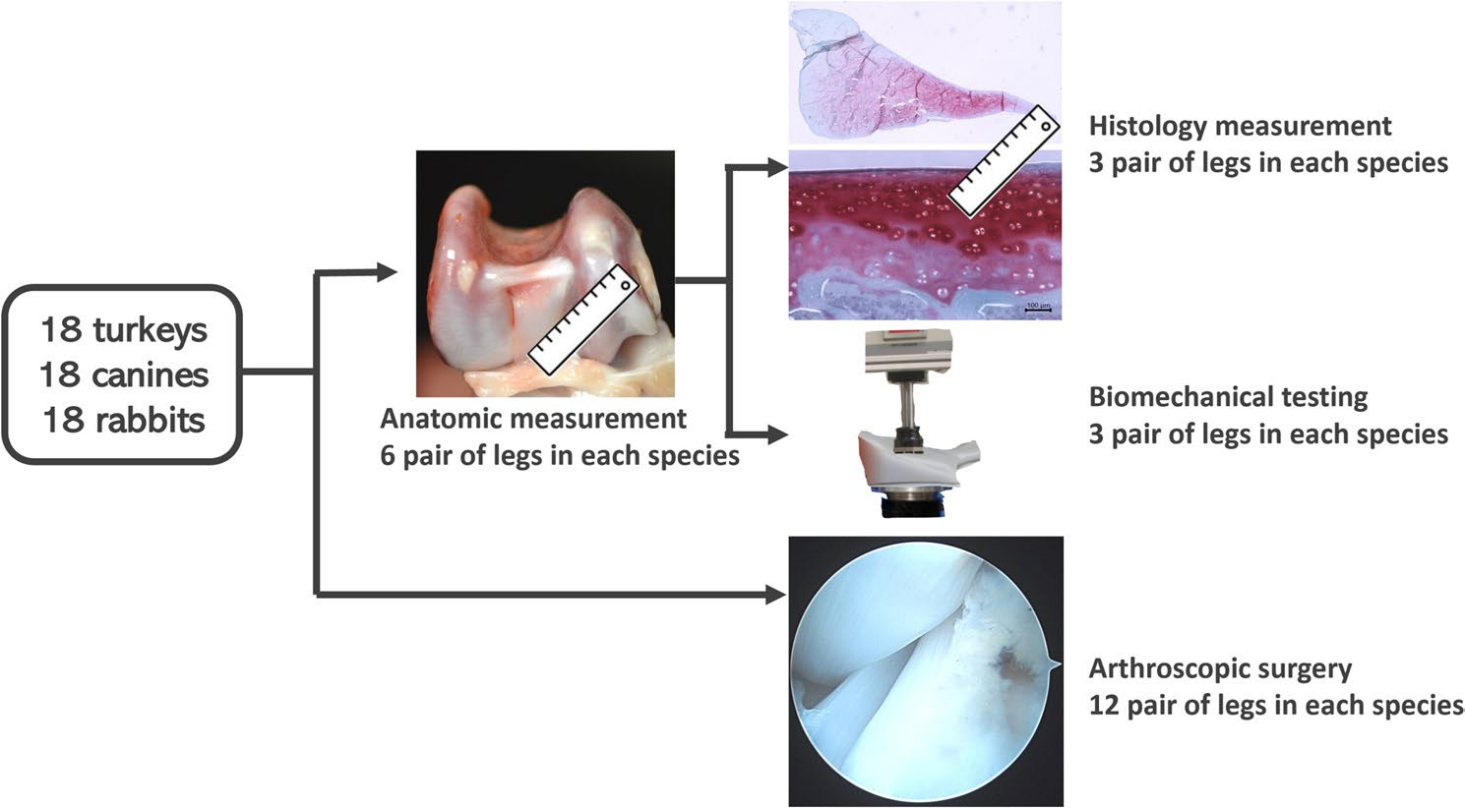
The schematic diagram of the animal experiment design. Knee samples were harvested from fresh turkeys (*n* = 18, 18 pairs), canines (*n* = 18, 18 pairs), and rabbits (*n* = 18, 18 pairs). Six pairs of legs in each species were used for anatomical measurements, histological analysis, and biomechanical studies. The remaining twelve pairs of legs in each species were used for arthroscopic surgery

For animals used in the basic study, each leg was dissected for anatomical measurements. Subsequently, six pairs of legs in each specimen were assigned to histological and biomechanical examinations **(**Fig. 1**).** All specimens were frozen at the time of collection and maintained at − 80 °C until the time of dissection and analysis. One day before dissection, they were thawed at room temperature.

### Tissue dissection and anatomic measurements

In preparation for the dissection of the turkey knee specimen, the skin and feathers were carefully removed. The surrounding muscle of the knee joint was meticulously dissected, leaving only the extra-articular ligament intact. Using a vernier caliper, precise measurements were taken to determine the midpoint width and length of both the medial collateral ligament (MCL) and the lateral collateral ligament (LCL). After measuring the size of the extra-articular ligament, an arthrotomy was performed from the medial side, starting with a supra-patellar incision extending to remove the patella and the patellar tendon. This removal not only facilitated the measurement of the patella’s size but also provided a clear view of the anterior joint structures. With this enhanced perspective, it became possible to observe and measure the cranial cruciate ligament and caudal cruciate ligament under hyperflexion, corresponding to the anterior and posterior cruciate ligament in humans.

Following that, the collateral ligaments and cruciate ligaments were removed, and the femur was detached to facilitate a clear visualization of the bilateral menisci. Measurements were taken for the height and width of the femoral condyle, trochlear, and intercondylar notch, as well as the midpoint width and length of medial and lateral menisci. Subsequently, the menisci were excised, uncovering the tibial plateaus and their articulation with the fibula. The width of the tibial plateaus was subsequently measured. Each structure was measured three times, and the median value was reported. This dissection process was also performed on the knee specimens of canines and rabbits.

In addition to the direct measurements, all values were normalized by the width of the tibial plateau, except in turkeys, where the femur articulates with both the tibia and fibula. For turkeys, the measurement was normalized to the width of the combined tibia and fibula. This normalization process resulted in a ratio known as the Tibial Index, which represented the relationship between the width of the tibial plateau (or tibia-fibula plateaus in turkeys) and the size of the measured structures. In the subsequent comparative statistical analysis, both the measured values and the values normalized to the Tibial Index were utilized.

### Histological examination

The histological examination encompassed the medial meniscus and cartilage of the femoral condyle. Regarding the medial meniscus, the specimens were fixed in a 10% neutral buffered formalin solution before being embedded in paraffin following excision. Subsequently, transverse blocks were selectively trimmed from the middle portions of each meniscus and stained with hematoxylin/eosin and Safranin O. To determine the cross-sectional size of the menisci, precise measurements were taken of the area (mm^2^) occupied by meniscal tissues. Additionally, the area (mm²) of Safranin O-positive tissue was measured and normalized to the total meniscal tissue area (mm²).

For cartilage histology, the distal femur was excised and fixed in 10% formalin, followed by decalcification with ethylenediaminetetraacetic acid. Specimens were then sectioned in the sagittal plane through the central region of the distal medial condyle. These sections were then stained with Safranin O. A point-to-point measurement from the articular surface to the subchondral bone plate was conducted to determine cartilage thickness. The ratio of articular cartilage to calcific cartilage was calculated. The ImageJ software (National Institute of Health, Bethesda, MD) was used to measure thickness and area.

### Biomechanical testing

The biomechanical testing focused on the medial menisci, and an unconfined compression test was applied to the medial menisci, a custom mechanical testing system equipped with a 3.0-mm-diameter plane indenter. The indenter was displaced at a constant rate of 0.1 mm/sec, while force and displacement data were sampled at 50 Hz. In the case of the canine and turkey specimens, the middle part of the menisci was pre-cut into a 4 mm x 4 mm x 1 mm piece, and for the rabbit, the piece used was as large as possible. To ensure stability during testing, sandpaper was placed between the menisci and the base, preventing the tendon from sliding on the base.

The mechanical test data was processed using LabVIEW (LabVIEW2012; National Instruments, Austin, USA), resulting in the creation of a stress-strain curve. The compressive elastic modulus was estimated by determining the slope of a line fit to the stress-strain data. To represent each meniscus, the median value of the compressive modulus from the three measurements was calculated.

### Arthroscopic exploration

Twelve pairs of legs in each animal species were used for arthroscopic surgery. Prior to the procedure, the specimens were allowed to thaw outside the freezer for approximately 24 h. In preparation for exploration, the skin was carefully removed, and the surrounding muscle proximal to the halfway point of the thigh was trimmed to facilitate access to the proximal femur. Following that, the proximal half of the femur was securely held in place using a custom-made clamp. Standard arthroscopy instruments, including a 2.7-mm arthroscope with a 30-degree visual angle, a 2.5-mm hook probe, a 3.5-mm arthroscopic shaver (Stryker aggressive cutter 375-638-000), a 3.5-mm arthroscopic coagulator (Stryker SERFAS energy 279-351-100), were employed. An arthroscopy pump system was utilized to maintain an intra-articular pressure of 30 mm Hg.

With the knee positioned at approximately 90 to 100 degrees of flexion, the patella and tibial tuberosity were palpated. The anterolateral and anteromedial portals were then marked slightly proximal to the tibial tuberosity and beside the patellar tendon, confirmed by testing with an 18-gauge needle. The anterolateral portal was created using an 11-blade scalpel, and the arthroscope was inserted into the joint, directed proximally and medially with knee extension to inspect the suprapatellar pouch and patellofemoral articulation. With the knee joint passive range of motion, the patella tracking in trochlear groove could be observed. The joint was subsequently insufflated. Then the scope was advanced inferiorly, posteriorly and medially with gentle knee flexion to 90 degrees to navigate behind the infra-patellar fat pad. Under direct visualization with spinal needle localization, the anteromedial portal was established using the same 11-blade scalpel.

Following the removal of the fat pad, further arthroscopic examination was performed, allowing for thorough exploration. The arthroscope was carefully directed towards the medial compartment, providing a clear view of the medial femoral condyle, anterior horn, and body of the medial meniscus. The arthroscope was then rotated to gain a comprehensive view of the intercondylar notch, facilitating the visualization of both the caudal cruciate ligament and cranial cruciate ligament [22]. Continuing the procedure, successful visualization of the lateral compartment was achieved. Similar to certain mammals like the canine [23], rabbit [24] and minipig [15], the extensor digitorum longus tendon was found to be intra-articular, originating from the craniolateral aspect of the lateral femoral condyle. By retracting the tendon, the lateral femoral condyle and meniscus were uncovered, offering a clear view.

### Evaluation of performing arthroscopy

**Fig. 2:**
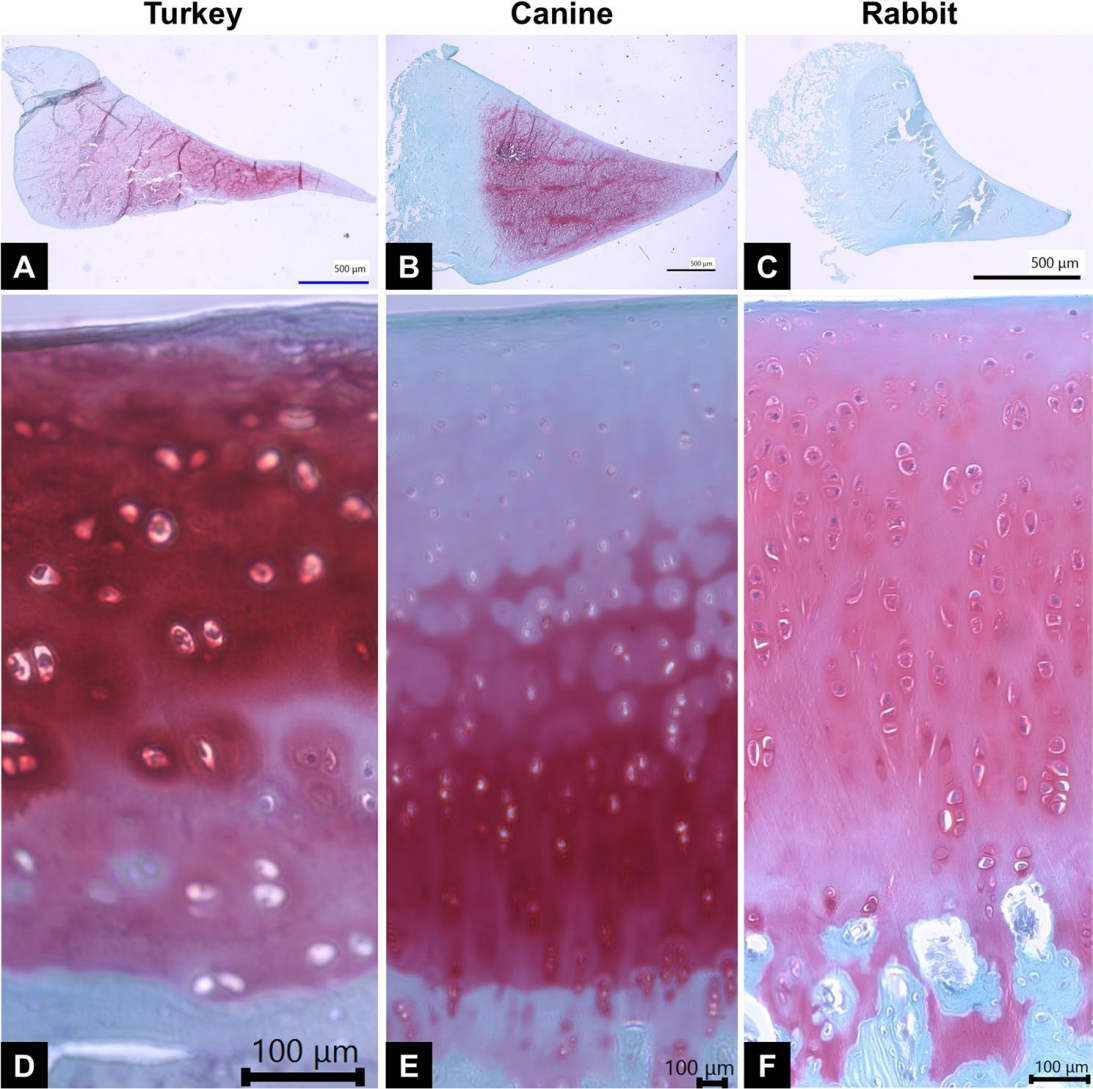
Gross view and arthroscopic findings in three species. The arthroscopic image and gross view of the right knee in **A** turkey, **B** canine and **C** rabbit respectively. **D**-**G** The arthroscopic findings in turkey knee: **D **The arthroscopic image obtained during the excision of cranial cruciate ligament (as anterior cruciate ligament in humans, ACL). **E**The arthroscopic image obtained after excision of ACL (F)The arthroscopic image obtained during medial meniscectomy. **G **The arthroscopic image obtained after total medial meniscectomy. 1, caudal cruciate ligament (as posterior cruciate ligament in humans); 2, lateral femoral condyle; 3, cranial cruciate ligament (as anterior cruciate ligament in human); 4, medial meniscus; 5, lateral meniscus

Four orthopaedic sports medicine surgeons (the first two, fourth, and fifth author in the author list) were enlisted to partake in arthroscopic exploration of the turkey knee. Prior to the surgery, each participant dissected a cadaveric specimen while performing anatomical measurements to familiarize themselves with the overall anatomy. Subsequently, each surgeon performed arthroscopic exploration, cranial cruciate ligament transection, and total medial meniscectomy **(**Fig. 2**)**. Finally, the total procedure duration was recorded. During arthroscopic exploration, each participant completed a set of 10 tasks. This checklist of 10 tasks was devised by Koehler et al. for evaluating diagnostic knee arthroscopy skills on cadaveric models **(**Table 1**)** [25].

**Table 1:** The checklist of the 10-task arthroscopic exploration which devised by Koehler et al. [25]

1 Inspect suprapatella pouch
2 Evaluate patella and patellofemoral articulation
3 Evaluate patella tracking in trochlear groove
4 Inspect medial gutter
5 Inspect and probe medial femoral condyle and tibial plateau
6 Inspect and probe anterior, middle, and posterior medial meniscus
7 Inspect and probe cranial/caudal cruciate ligament (as the ACL/PCL in human)
8 Inspect and probe lateral femoral condyle and tibia plateau
9 Inspect and probe anterior, middle, and posterior lateral meniscus
10 Inspect the lateral gutter, popliteus tendon and recess

### Statistical analysis

Due to the significant variance between each specimen, we focused our statistical analysis on the normalized anatomic measurement, biomechanical, histological data and surgical time. An a priori power analysis was performed using G*Power to compare surgical time across the three groups. Based on pilot study data, where each surgeon operated on two specimens per group (*n* = 2 per surgeon per group), with an alpha level of 0.05 and a power of 0.80, the required sample size for statistical comparison was estimated to be approximately six per group. In the main study, each group was operated on by four surgeons, with each surgeon performing procedures on six specimens, resulting in a total of 24 specimens per group. This sample size was considered sufficient to achieve the study’s primary objective while accounting for potential attrition. To determine differences of anatomic measurement, histologic examination and biomechanical tests between groups, we conducted an analysis of variance (ANOVA), with the significance level set at *p* < 0.05 for the overall ANOVA. The time taken to complete arthroscopic procedures among species and surgeons was analyzed using a two-way ANOVA, followed by multiple comparisons using Tukey’s honestly significant difference (HSD) test. All these calculations were efficiently carried out using the software package SPSS (Version 11.0, SPSS Inc., Chicago, Illinois).

## Results

### Observation

All observations presented herein describe major differences noticed visually during dissection of the turkey knee as they relate to the canine and rabbit knee with an emphasis on the overall shape of bony structure and soft tissue.

In the case of extra-articular structures, the patella was found in the suprapatellar pouch and moves along the patellar groove during knee joint motion in all three animals. The turkey patella is wider than it is tall, and similarly, the tibial tuberosity is more prominent in turkeys. In comparison to canines and rabbits, when normalized to the width of the bony structure, all extensor components, including the patella, patellar tendon, and tibial tuberosity, appear wider. This wide extensor structure poses a challenge in exposing the intra-articular structures without transection of the patellar tendon.

Regarding the intra-articular structures, there are three significant differences between turkeys and canines as well as rabbits. First, in the turkey femoral condyle, the trochlea is wider and deeper, potentially engaging the wide patella **(**Fig. 2**)**. Secondly, the arrangement of cruciate ligaments in turkeys differs from that in canines and rabbits. The caudal cruciate ligament, which spans from the anterior femoral inter-condyle to the medial-posterior portion of the tibia plateaus, functions as the posterior cruciate ligament (PCL) does in humans. However, it is located anterior to the cranial cruciate ligament, which attaches from the posterolateral femoral condyle to the anterior tibial plateau and acts as the anterior cruciate ligament (ACL) (No. 1 in Fig. 2A, B and C**)**. Thirdly, the femur is articulated with both the tibia and fibula. The articulation between the lateral femoral condyle and the fibular head gives the lateral meniscus an irregular shape, distinct from the crescent-shaped or circular form.

### Anatomic measurements

The values of the anatomic structure around knee and those normalized with tibia-fibula width were listed in Table 2. After measured values normalized by tibia-fibula width, there were still significant differences in the width and height of the patella, widths of MCL and LCL, length and width of ACL, width of PCL, width of lateral meniscus, femoral condylar height and width, as well as trochlear width and depth.

**Table 2:** The measurements of the knee joint between the three species. Presentation of the mean. MCL, medial collateral ligament; LCL, lateral collateral ligament; ACL, anterior cruciate ligament; PCL, posterior cruciate ligament. *Reach significant difference after analysis of variance

Characteristics	Measured value (mm)			Tibia index (measured value normalized by tibia-fibula width)			*p*- value for Tibia-index
	Turkey (*n* = 12)	Canine (*n* = 12)	Rabbit (*n* = 12)	Turkey (*n* = 12)	Canine (*n* = 12)	Rabbit (*n* = 12)	
Patella							
Width	22.38	12.25	6.00	0.72	0.30	0.33	< 0.001*
Height	12.78	19.92	10.00	0.41	0.49	0.55	< 0.001*
MCL							
Length	35.25	44.17	22.37	1.13	1.09	1.24	0.132
Width	8.92	7.17	3.17	0.29	0.18	0.17	< 0.001*
LCL							
Length	30.92	37.00	17.83	0.99	0.91	0.98	0.586
Width	6.54	4.83	2.75	0.21	0.12	0.15	< 0.001*
ACL or Cranial cruciate ligament							
Length	14.98	18.25	6.92	0.48	0.45	0.38	0.009*
Width	3.08	5.17	2.50	0.10	0.13	0.14	0.003*
PCL or Caudal cruciate ligament							
Length	14.83	19.33	7.33	0.47	0.45	0.41	0.086
Width	3.96	3.50	2.83	0.13	0.09	0.16	< 0.001*
Medial menisci							
Length	16.71	19.42	9.17	0.53	0.48	0.50	0.101
Width							
Anterior	5.75	6.58	3.25	0.18	0.16	0.18	0.247
Middle	3.71	6.08	2.33	0.12	0.15	0.13	0.066
Posterior	5.21	6.67	2.92	0.17	0.16	0.16	0.828
Lateral menisci							
Width	7.29	7.17	3.50	0.23	0.18	0.19	< 0.001*
Intraarticular bony structure							
Femoral condylar width	29.88	38.50	17.83	0.96	0.95	0.98	0.854
Femoral notch width	6.08	8.67	3.33	0.19	0.21	0.18	0.174
Femoral condylar height							
Medial	25.13	41.50	19.50	0.80	1.02	1.07	< 0.001*
Lateral	23.67	39.58	18.75	0.76	0.95	1.03	< 0.001*
Trochlear width	10.83	7.33	3.08	0.35	0.18	0.17	< 0.001*
Trochlear depth	9.48	2.41	1.25	0.30	0.059	0.07	< 0.001*
Tibia width (Tibia-fibula width in Turkey)	31.25	40.58	18.25	1.00	1.00	1.00	-

### Histology

**Fig. 3:**
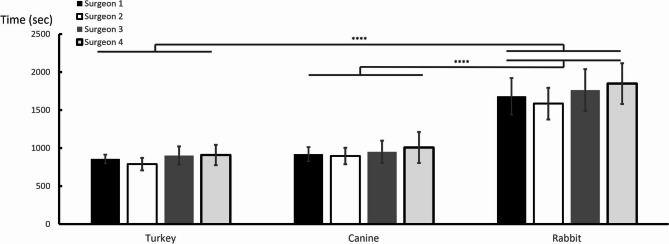
The histological presentation of medial meniscus and femoral distal medial condyle cartilage. **A**~**C** safranin O stain in the medial meniscus of turkey, canine and rabbit separately, scale bar = 500 μm. **D**~**F **the Safranin O stain in distal femoral medial condyle of turkey, canine and rabbit respectively, scale bar = 100 μm

The Safarin O positive area in the medial meniscus and the articular cartilage thickness were significantly different among the three species **(**Fig. 3; Table 3**)**. The Safarin O positive area was 31.72 ± 13.60, 26.86 ± 17.43, 0.32 ± 0.16 in turkeys, canines and rabbits, respectively (*p* < 0.001). The thickness of the articular cartilage was 438 ± 35, 1036 ± 67, 621 ± 82 μm in turkeys, canines and rabbits, respectively (*p* < 0.001). The thickness ratio of the articular cartilage to calcified cartilage was 2.1 ± 0.39, 4.3 ± 0.85, 3.2 ± 0.62 in turkeys, canines and rabbits, respectively (*p* < 0.001).

### Biomechanical testing

The stiffness in the medial meniscus showed no significant difference between the three species (Table 3). The stiffness was 26.01 ± 3.23, 26.74 ± 1.81, and 29.16 ± 1.60 in turkeys, canines and rabbits, respectively (*p* = 0.079).

### The duration in arthroscopic exploration and procedure

**Fig. 4:**
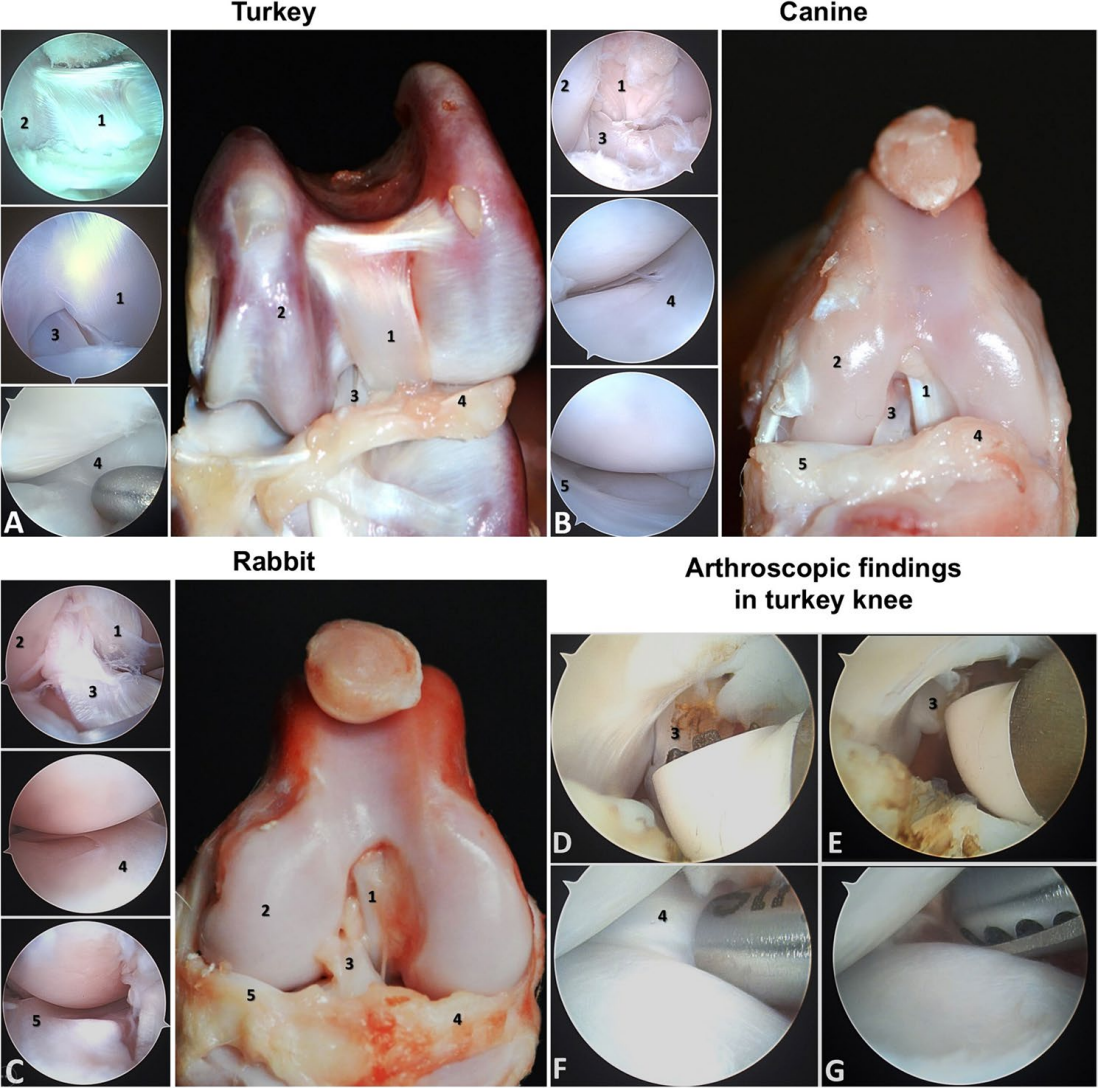
Arthroscopic operation time in different surgeons and species. The operation time was obtained after completing 10-task arthroscopic exploration, cranial cruciate ligament transection (as anterior cruciate ligament in humans), and total medial meniscectomy. There was no significant difference between the surgeons in the same species. The arthroscopic operation time in rabbit was significantly longer than turkey and canine whereas there was no significant difference between turkey and canine after multiple comparisons in all surgeons (*p* = 1.00, < 0.001, < 0.001 in turkey vs. canine, rabbit vs. turkey, rabbit vs. canine separately). ****p < 0.001

There was no significant difference between the surgeons in the same species in the time taken to complete the arthroscopic exploration tasks, cranial cruciate ligament excision and total medial meniscectomy **(**Fig. 4**)**. In turkeys, the operation time was 857 ± 56.6, 791 ± 82.3, 902 ± 119, 909 ± 134 in surgeon 1, 2, 3, and 4, respectively which showing no significant difference between the surgeons (the lowest *p*-value was 0.08 in surgeon 2 compared with surgeon 4 after post hoc analysis). When comparing the arthroscopic operation time in different species, the time taken in rabbits was significantly longer than turkeys and canines whereas there was no significant difference between turkey and canine after multiple comparisons in all surgeons (*p* = 1.00, < 0.001, < 0.001 in turkey vs. canine, rabbit vs. turkey, and rabbit vs. canine, respectively) **(**Fig. 4**).**

**Table 3:** Biomechanical properties and histologic analysis of the medial meniscus of each species; histologic examination of the distal medial condyle cartilage of each species. Data presented as mean ± SEM. *Reach significant difference after analysis of variance

	Turkey	Canine	Rabbit	*p*-value
Meniscus				
Stiffness (N/mm)	26.01 ± 3.23	26.74 ± 1.81	29.16 ± 1.60	0.079
Cross-section (mm2)	2.47 ± 0.44	4.62 ± 1.04	0.90 ± 0.39	< 0.001*
Safranin-O positive area (%)	31.72 ± 13.60	26.86 ± 17.43	0.32 ± 0.16	< 0.001*
Medial femur condyle cartilage				
Cartilage thickness (µm)	438 ± 35	1036 ± 67	621 ± 82	< 0.001*
The ratio of calcified cartilage to calcific cartilage	2.1 ± 0.39	4.3 ± 0.85	3.2 ± 0.62	< 0.001*

Biomechanical properties and histologic analysis of the medial meniscus of each species.

Histologic examination of the distal medial condyle cartilage of each species

Data presented as mean ± SEM.

*Reach significant difference after analysis of variance

## Discussion

The study not only examined the anatomical, biomechanical, and histological properties of the turkey knee but also investigated the feasibility of performing arthroscopic surgery on the turkey knee compared with other commonly used animal species, canines and rabbits. Despite certain anatomical distinctions and differences in cartilage thickness between the turkey knee and those of rabbits or dogs, they share a common feature in the medial meniscus. Additionally, the meniscus of turkeys exhibits comparable biomechanical and histological characteristics to those found in canines. Moreover, experienced orthopedic surgeons could perform arthroscopic surgery on the turkey knee without a significant longer time compared with canines and rabbits.

As a medium-sized avian species, the turkey may possess certain attributes that could potentially make it a suitable candidate for knee-related research. Primarily, the overall size of the turkey and its knee fall between those of dogs and rabbits, thus encompassing the advantages inherent in both small and large animal models. In its role as a small animal model, the turkey offers cost-effectiveness and rapid growth [17, 26]. Conversely, its status as a large animal model allows for routine diagnostic imaging, arthroscopic interventions, and comprehensive postoperative management, particularly relevant to OA research. Consequently, the turkey model proves invaluable not only for comprehending the pathogenesis and pathophysiology of the ailment, but also for gauging treatment responses. Furthermore, due to its bipedal nature, turkeys and humans exhibit analogous stride lengths and duty factors during the gait cycle [27]. The adaptations employed by both turkeys and humans during swing-leg motion when navigating uneven terrain, such as handling drops and descending inclines, as well as overcoming obstacles and ascending steps, appear remarkably similar [28]. In contrast, common OA animal models predominantly involve quadrupeds, leading to distinct compensatory gait adjustments compared to bipedal humans. The impact of these adaptations on gait varies depending on the affected joint.

Unfortunately, some significant distinctions in the knee structure exist between turkeys and mammals, and these differences impose certain limitations on utilizing turkeys as an animal model. Firstly, the deeper trochlear groove in the femoral condyle and the broader patella complicate the parapatellar approach. Joint exploration without patellar tendon transection becomes unfeasible, necessitating the use of arthroscopic techniques for assistance. A pronounced patellar groove may limit its suitability for studying patellofemoral joint pathologies, including patellofemoral OA and patellar instability. Secondly, the distinct orientation of cruciate ligaments in turkeys impedes their suitability as an appropriate model for studying issues related to the ACL or PCL. Lastly, due to the irregularly shaped lateral meniscus and its unique articulation between the femoral condyle, tibia, and fibula, turkeys are unsuitable for simulating the human lateral meniscus in research.

By meticulously assessing the anatomical, histological, and biomechanical properties of the turkey knee, we have found that the turkey provides an alternative avenue for studying the medial meniscus. In terms of anatomy, we have observed a similarly circular shape and cross-section shared by both turkeys and humans. The tibial index for the length of the turkey’s medial meniscus is 0.54 ± 0.06, which is more similar to that of humans (0.57 ± 0.04) [8], compared to figures from dogs (0.47 ± 0.03) and rabbits (0.50 ± 0.03). Similarly, the tibial index for the width of the turkey’s medial meniscus (0.12 ± 0.02) closely resembles that of humans (0.14 ± 0.01). This suggests that the size of the turkey’s medial meniscus in relation to the knee joint is comparable to that of humans. Regarding histology, the safranin O staining intensity in human menisci tends to be inconsistent, possibly due to age-related degeneration [29, 30]. However, we have found comparable safranin O staining intensity in both turkey and dog menisci, with nearly one-third of meniscal tissue exhibiting a positive safranin O response. Biomechanically, the turkey meniscus exhibits similar stiffness to that of dog and rabbit menisci under compressive testing.

When examining the histology of the distal femur medial condylar cartilage, it was observed that the thickness of the cartilage in turkey (438 ± 35 μm) was thinner compared to that of canines (1036 ± 67 μm) or rabbits (621 ± 82 μm). This suggests that turkey could potentially serve as an ideal model for studying OA due to its exceptionally thin cartilage, even thinner than that of rabbits. Additionally, even their larger size compared to small animals like rabbits but given to thinner cartilage, turkeys may eliminate some disadvantages such as slower OA progression typically seen in large animal models [31].

Several studies have investigated arthroscopic surgery in large animal model including porcine [15, 32–34], canine [35], bovine [36], and monkey [37]. The present study is the first to assess the feasibility of arthroscopic surgery in non-mammal animals, specifically turkeys. The 10-task checklist arthroscopic examination, cranial cruciate ligament excision and total medial meniscectomy adopted in the present study is a widely accepted tool for evaluating basic proficiency in arthroscopy [38]. Due to their smaller size and dimensions of femoral condylar width and tibia width, rabbits took longer during the arthroscopic procedure compared to turkeys and canines. When it came to turkey-sized animals, the use of a 2.7 mm scope, 3.5 mm shaver, and coagulator allowed surgeons to perform arthroscopic surgery more easily, resulting in similar operation times between turkeys and canines. The turkey model offers a valuable opportunity for studying arthroscopic knee surgery techniques, including arthroscopy training. Additionally, it serves as a model for traumatic knee injuries, such as cartilage and meniscus damage, while addressing the limitations of large animal models, including high costs, handling difficulties, and ethical concerns, particularly regarding public perception [31].

There are several limitations in the current study. First, the study focused only on the in vitro structure comparison. The potential for meniscal healing and chondrogenesis was not investigated. Nevertheless, this study demonstrated the feasibility of performing arthroscopy in turkey knees, which opens possibilities for further in vivo surgeries. Second, certain characteristics, such as range of motion, lower limb alignment, and the biomechanical properties of the ligaments, were not measured in this study due to the use of cadaveric lower limbs and limited instrumentation. Finally, human specimens were not included for comparative analysis. Consequently, we were unable to ascertain the degree of anatomical and histological similarity or difference between the species investigated in this research and human anatomy. However, comparisons were made with canine and rabbit specimens, both of which are commonly employed as large and medium animal models, respectively, in knee pathology research as indicated in previous publications [35, 39].

In conclusion, the turkey model shows promise as a valuable animal model for studying arthroscopic knee surgery and associated traumatic knee injuries. This model offers advantages such as comparable operation times to larger animal models like canines, and the potential for faster progression of OA due to its thinner cartilage compared to smaller animal models like rabbits. However, given the anatomical differences between the turkey and the human knee, careful evaluation is necessary to determine its suitability as a human simulator before conducting research.

## Supplementary Information

Supplementary Material 1.

## Data Availability

The datasets used and/or analysed during the current study are available from the corresponding author on reasonable request.

## Abbreviations

ACL: Anterior cruciate ligament
LCL: Lateral collateral ligament
MCL: Medial collateral ligament
OA: Osteoarthritis
PCL: Posterior cruciate ligament
