# Supplementary material for: Evaluating the Turkey as a novel model for arthroscopic knee surgery research: a comparative analysis with canine and rabbit
Source: BMC Musculoskelet Disord. 2025 Sep 2;26:843. doi: 10.1186/s12891-025-09098-6 (PMC12403518; doi:10.1186/s12891-025-09098-6)
Supplement: Supplementary file 1 — Supplementary Material 1. [file 12891_2025_9098_MOESM1_ESM.docx]

**Evaluating the Turkey as a Novel Model for Arthroscopic Knee Surgery Research:**

**A Comparative Analysis with Canine and Rabbit**

Kai-Lan Hsu^1,2*^, Po-Yen Ko^1,2*^, Katy L. Lydon^1^, Weihong Zhu^1^, Shunen Xu^1^, Ramona L. Reisdorf^1^, Chunfeng Zhao^1^

1. Mayo Clinic, Rochester, MN, USA

2. Department of Orthopedics, National Cheng Kung University Hospital, College of Medicine, National Cheng Kung University, Tainan, Taiwan

*KLH and PYK were contributed equally in this work.

**Funding:** This study was supported by a Mayo Orthopedic Research Review Committee grant

**Conflicts of interest:** The author(s) declared no potential conflicts of interest with respect to the research, authorship, and/or publication of this article.

**Correspondence and Reprint Requests to:**

Kai-Lan Hsu, MD, Department of Orthopedics, National Cheng Kung University Hospital, College of Medicine, National Cheng Kung University, Tainan, Taiwan

**Email:** [dulendulen@gmail.com](mailto:dulendulen@gmail.com)

Chunfeng Zhao, MD, Mayo Clinic, Rochester, MN, USA

**E-mail:** [Zhao.Chunfeng@mayo.edu](mailto:Zhao.Chunfeng@mayo.edu)

**Tel:** +1 5072660982

**Declarations:**

**Ethics approval and consent to participate:** This study utilized animal specimens obtained from previously approved ARRIVE-compliant studies. No live animals were used in this research. The use of these post-mortem specimens was approved by the Institutional Animal Care and Use Committee (IACUC) of Mayo clinic (approval number: A00007221-23; A00006768-22; A00006537-22). All procedures were conducted in accordance with ethical guidelines for the use of animal specimens in research.

**Consent for publication:** Not applicable

**Conflict of interest:** The authors declare that they have no competing interests

**Funding:** Internal funds were used to fund this study

**CRediT authorship contribution statement:**

Conceptualization: K.-L.H., P.-Y.K., C.-F.Z. ; methodology: K.-L.H., P.-Y.K., C.-F.Z.; software: K.-L.H., P.-Y.K., K.-L.L. C.-F.Z.; validation: P.-Y.K., K.-L.H., C.-F.Z.; formal analysis: P.-Y.K., K.-L.H., C.-F.Z.; investigation: K.-L.H., P.-Y.K., W.Z., S.X., R.-L.R., C.-F.Z.; resources: R.-L.R., C.-F.Z.; data curation: P.-Y.K., K.-L.H., C.-F.Z.; writing—original draft preparation: P.-Y.K., K.-L.H., C.-F.Z.; writing—review and editing: R.-L.R., C.-F.Z.; visualization: R.-L.R., C.-F.Z.; supervision: P.-Y.K., R.-L.R., C.-F.Z.; project administration: R.-L.R., C.-F.Z.; funding acquisition: R.-L.R., C.-F.Z.
